# A dynamic approach to support outbreak management using reinforcement learning and semi-connected SEIQR models

**DOI:** 10.1186/s12889-024-18251-0

**Published:** 2024-03-11

**Authors:** Yamin Kao, Po-Jui Chu, Pai-Chien Chou, Chien-Chang Chen

**Affiliations:** 1 Geometric Data Vision Laboratory, Department of Biomedical Sciences and Engineering, National Central University, Taoyuan City, Taiwan; 2 Division of Pulmonary Medicine, Department of Internal Medicine, Taipei Medical University Hospital, Taipei, Taiwan; 3 Division of Thoracic Medicine, Department of Internal Medicine, School of Medicine, College of Medicine, Taipei Medical University, Taipei, Taiwan

**Keywords:** COVID-19, Reinforcement learning, SEIQR model, Transport hub, Population-weighted density

## Abstract

**Background:**

Containment measures slowed the spread of COVID-19 but led to a global economic crisis. We establish a reinforcement learning (RL) algorithm that balances disease control and economic activities.

**Methods:**

To train the RL agent, we design an RL environment with 4 semi-connected regions to represent the COVID-19 epidemic in Tokyo, Osaka, Okinawa, and Hokkaido, Japan. Every region is governed by a Susceptible-Exposed-Infected-Quarantined-Removed (SEIQR) model and has a transport hub to connect with other regions. The allocation of the synthetic population and inter-regional traveling is determined by population-weighted density. The agent learns the best policy from interacting with the RL environment, which involves obtaining daily observations, performing actions on individual movement and screening, and receiving feedback from the reward function. After training, we implement the agent into RL environments describing the actual epidemic waves of the four regions to observe the agent’s performance.

**Results:**

For all epidemic waves covered by our study, the trained agent reduces the peak number of infectious cases and shortens the epidemics (from 165 to 35 cases and 148 to 131 days for the 5th wave). The agent is generally strict on screening but easy on movement, except for Okinawa, where the agent is easy on both actions. Action timing analyses indicate that restriction on movement is elevated when the number of exposed or infectious cases remains high or infectious cases increase rapidly, and stringency on screening is eased when the number of exposed or infectious cases drops quickly or to a regional low. For Okinawa, action on screening is tightened when the number of exposed or infectious cases increases rapidly.

**Conclusions:**

Our experiments exhibit the potential of the RL in assisting policy-making and how the semi-connected SEIQR models establish an interactive environment for imitating cross-regional human flows.

**Supplementary Information:**

The online version contains supplementary material available at 10.1186/s12889-024-18251-0.

## Introduction

Containment measures to limit mobility and implement social distancing are proven effective in slowing the spread of COVID-19 [1]. However, these efforts also reduce economic activity [2] and have led to dramatic impacts on global and domestic economies [3, 4]. Instead of growing a projected 2.5 percent, the global GDP of 2020 shrank by 3.3 percent [5, 6]. This emphasizes the challenges and importance of dynamic disease modeling for governments to make policies [7].

Mathematical models play a key role in decision-making [8]. Various computational methods have supported COVID-19 management, including statistical, compartmental, spatial metapopulation, and agent-based network models, as well as machine learning (ML) [9–12]. Statistical and ML methods learn patterns from time-series data for short-term trend projection and forecasting [13–16]. Compartmental models of Susceptible-Infected-Removed (SIR) and Susceptible-Exposed-Infected-Removed (SEIR) are widely used to study the dynamics and spread of diseases [17, 18]. In these models, the population is confined, isolated from other populations, and homogeneous. Spatial metapopulation models use geographic data to partition the populations into subpopulations, which are connected by human flow matrices and governed by respective SEIR models [19]. Hence, heterogeneous mobility patterns among subpopulations can be adequately reflected. These models are beneficial at the initial phase of the outbreak when disease spread is significantly driven by mobility; however, they are less helpful in modeling the effect of containment on disease spread within or across subunits [9].

Agent-based network models further delve into disease spread within a population by simulating interactions at the individual level [20]. It is important to evaluate not only the benefits of specific inventions but also their economic aspects [21]. Researchers have used this approach with extended SIR models to simulate the impact of government policies on reducing disease spread while maintaining economic activities. Nishi et al. defined 2 network intervention strategies for people’s group activities [22]. The first strategy is to split a group into 2 subgroups (“dividing groups”). For example, one subgroup of customers can only go to the grocery in the morning, while the other subgroup can only go in the evening. The second strategy is to redistribute members across different groups evenly (“balancing groups”). For example, some of the customers who go to the popular store are redirected to the less popular one. Their results show that the dividing strategy significantly suppresses the transmission. Moreover, the implementation of both dividing and balancing can effectively keep the effective reproduction number around 1 (i.e., having the disease spread under control). Shami and Lazebnik established a model that links epidemiological dynamics, pathogen mutation, policy on sequencing tests, and economic dynamics and applied deep neural networks to determine the optimal policy [23]. Their simulation demonstrates that detecting new strains is effective; however, proper implementation is essential for better epidemiological and economic outcomes and can be supported by artificial intelligence solutions regarding the testing subset and sample size. Lazebnik et al. developed a model consisting of epidemiological, spatial, and economic sub-models to examine the effect of intervention policies on industry production and supply [24]. The interventions involve worker separation (capsules vs. work-from-home) and vaccination. The capsule intervention is to randomly divide workers of each company into 2 groups to have them work alternatively; while the work-from-home intervention is to randomly select a percentage of workers of each company to work from home. It is found that the effectiveness of decreasing economic loss in order are vaccination, work-from-home, and capsules. Although agent-based models incorporate interventions or behavioral changes, they are data-intensive and computationally expensive [9].

Reinforcement learning (RL) creates an artificial environment for a virtual agent to interact with and take actions to maximize the cumulative reward based on the Markov Decision Process [25]. The agent learns to make optimal decisions via feedback from the environment instead of ground truths. Hence, RL is especially beneficial in situations with no gold standard and has extensively touched many fields, including healthcare [26–31]. Ohi et al. applied RL to explore the optimal control of epidemic spread [32]. They used 100%, 75%, and 25% as the permissible values of daily movement to represent discrete actions of no lockdown, social distancing, and lockdown in an SEIR environment. Although their experiments show that the trained agent balances epidemic control and the economic situation, the usage of discrete types of action limits the domain of activity mapping.

Our motivation is to establish a dynamic migration system, which allows us to observe the interaction between policies and disease transmission, and our study aims to establish an RL algorithm that (1) expands a typically isolated SEIR model to an open system that accommodates multiple regions; (2) imitates human activities by mapping individual movements into a continuous domain; (3) constraints moving capability with screening and quarantine mechanisms; (4) allows traveling through transport hubs underlying the migration mechanism simulated using population-weighted density; (5) mitigates disease spread while maintaining economic activities; and (6) provides insight on action timing.

## Methods

### Data description

Data of daily confirmed cases at the prefecture level for the study period from 25 January 2020 to 1 October 2021 were obtained from the open dataset provided by Toyo Keizai Online [33] (see Additional file 1). Four prefectures –Tokyo, Osaka, Okinawa, and Hokkaido – were selected for experiments due to their geographic relationship. Population and area data of these prefectures and their administrative subregions were extracted from the report of the 2015 population census [34] and 2020 planimetric reports [35] (see Additional file 2) for deriving population density (PD) and population-weighted density (PWD) (Table 1):

1 $${\text{PD}}=\frac{{\text{Population}}}{{\text{Area}}},$$

and

**Table 1:** Population density and population-weighted density

Parameter	Tokyo	Osaka	Okinawa	Hokkaido
Population (ppl)	13,515,271	8,839,469	1,433,566	5,381,733
Area (km^2^)	2,190.93	1,905.14	2,281.12	83,424.31
PD (ppl/km^2^)	6,169	4,640	628	69
PD percentage	53.6%	40.3%	5.5%	0.6%
PWD (ppl/km^2^)	13,032	8,359	3,549	1,615
PWD percentage	49.1%	31.5%	13.4%	6.0%

2 $${\text{PWD}}=\frac{\sum \left({\text{Population}}\times {\text{PD}}\right)}{\sum {\text{Population}} }.$$

### COVID-19 SEIQR environment

The SEIR compartmental model has been an essential tool for projecting the dynamics and spread of infectious diseases, including COVID-19 [9]. In an SEIR model, a population is divided into 4 mutually exclusive states, which are susceptible ($S$), exposed but not yet contagious ($E$), infectious ($I$), and recovered or deceased ($R$), each representing a fraction of the population. To incorporate the mechanisms of screening and quarantine, we add a quarantined (Q) state.

**Fig. 1:**
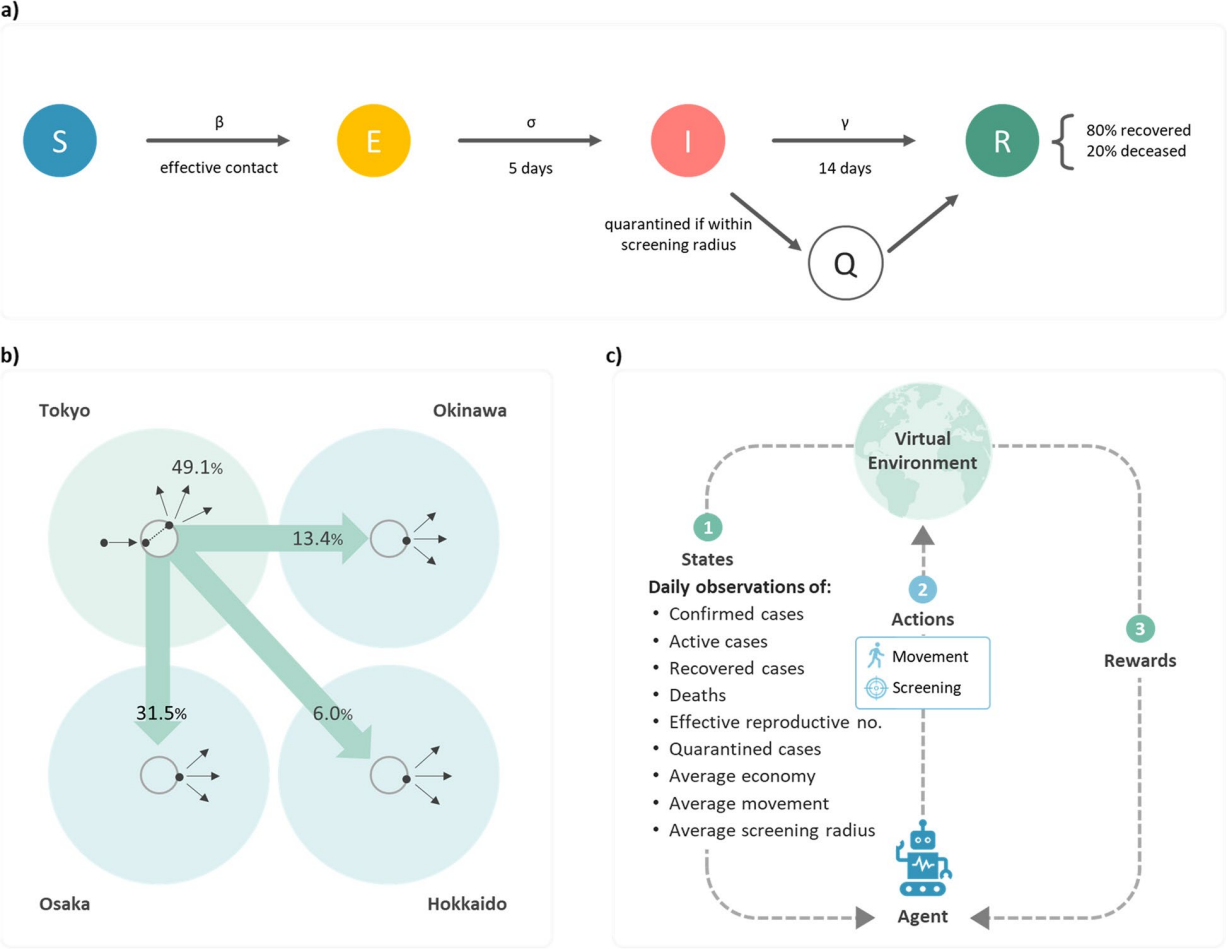
RL environment design and interactions with RL agent. **a** Transition of the SEIQR model. **b** Population flow governed by PWD via the transport hub, using Tokyo as an example. When inter-regional traveling occurs, the passenger will randomly appear at the edge of the destination’s transport hub and then keep moving. **c** Interactions between the Agent and the Environment

Figure 1a illustrates the transition of our SEIQR model: a susceptible individual transits to the exposed state after having effective contact with an infectious case, becomes infectious after 5 days [36], and remains infectious for 14 days before recovery or death. An infectious individual will be quarantined if within the screening radius. The survival rate is adjusted to 80% instead of 98% because the population size is only 500 in each RL environment due to the limitation of computational capability. From the perspective of computational modeling, if the fatality rate was set to 2%, deaths could not be generated reasonably in simulations. Therefore, we adjust the fatality rate to 20%, so that 10 deaths for each region on average can be observed in the RL environment. The proposed SEIQR model and the well-trained agent will mutually counteract the number of predictive deaths. A system of ordinary differential equations expresses the transition rates between states:

3 $$\frac{dS}{dt}=-\beta \frac{SI}{N},$$

4 $$\frac{dE}{dt}= \beta \frac{SI}{N}-\sigma E,$$

5 $$\frac{dI}{dt}=\sigma E-\gamma I-\frac{dQ}{dt},$$

and

6 $$\frac{dR}{dt}= \gamma I+\frac{dQ}{dt}.$$

The parameter $N$ is the population that equals the sum of $S$, $E$, $I$, $Q$, and $R$. The coefficients $\beta$, $\sigma$, and $\gamma$ represent contact, transmission, and recovery rates, respectively. The daily quarantine rate $dQ/dt$ is generated from the RL algorithm and bounded by formulas and model coefficients.

In our study, an SEIQR-RL environment (hereinafter Environment) consists of 4 regions corresponding to the 4 prefectures. For visualization, these regions are displayed by 4 circles with a radius of $50\sqrt{2}$ pixels and connected via transport hubs (5-pixel radius) placed at the centers of the circles. A synthetic population of 500 individuals is spatially distributed to these regions proportional to PWD. Each subpopulation has its own SEIQR model.

Whether a susceptible individual is exposed after having contact with an infectious individual depends on the effectiveness of contact, which is determined by transmission distance and infection probability. Our study simplifies it by defining only the effective transmission radius (${r}_{t}$) and letting the probability equal 1. That is, whenever a susceptible individual is within ${r}_{t}$ of an infectious case, the individual is exposed. The hyper-parameter ${r}_{t}$ is obtained through simulations in the conventional COVID-19 SEIR models and is defined as 0.11 pixels. An additional set of figures shows this in more detail (see Additional file 3).

In the simulated COVID-19 Environment, individuals make hourly movements to generate economy unless quarantined. These movements either take place within the initial region or between regions. Inter-regional traveling is also governed by PWD to ensure consistent subpopulations during simulations. For example, when an individual in Tokyo enters its transport hub, the chances of staying in Tokyo or traveling to Osaka, Okinawa, or Hokkaido are in proportion to PWDs, which are 49.1%, 31.5%, 13.4%, and 6.0%, respectively (Fig. 1b).

Massive vaccination was initiated in April 2021, and a substantial portion of the population was completely vaccinated by the end of the 5th wave (see Additional file 4). Although the effect of vaccination can be modeled by reducing the transmission radius, it is not included in our study because its effectiveness cannot be adequately translated into the model without increasing computational complexity.

### RL agent design and training

Our RL agent (hereinafter Agent) has two types of actions (outputs) – movement and screening. These actions are in a continuous domain with moving distances ranging from 1 to 5 pixels (1 = full action, 5 = none) and screening area with radiuses ranging from 0 to 10 pixels (10 = full action, 0 = none). Actions are executed daily at the prefecture level in the Environment. Each region's screening area overlaps its transport hub and shares the center.

To train the Agent, we use a deep neural network structured with bidirectional long short-term memory (LSTM) [37] layers for learning from consecutive time serial data. Asynchronous advantage actor-critic (A3C) [38] with 18 workers is used to increase data diversity and update speed by parallel computing. Proximal policy optimization (PPO) [39] and generalized advantage estimation (GAE) [40] are also used to reduce variances in training hence providing more reliable and accurate estimates.

Figure 1c illustrates how the Agent learns the best policy through interacting with the Environment. In each training round, the RL Environment provides daily states of the 4 prefectures for 15 consecutive days to the Agent, including the numbers of confirmed cases, active cases, recovered cases, deaths, quarantined cases, effective reproductive numbers, average movements, average screening radiuses, and average economies. The first 6 states are generated by the SEIQR models. Average movements and screening radiuses are collected from the actions assigned in the previous round, and the average economies are contributed by unquarantined individuals. These inputs are divided by either populations or respective maximums to remove the units. The Agent then grants daily actions on movement and screening for the 4 prefectures to the RL Environment based on these states. Finally, the Environment gives feedback to the Agent regarding the actions with a reward generated by the reward function. The Agent estimates the possible actions for the next round according to the reward values.

In our experiment, increases in the average economy are designed to generate positive rewards. In contrast, increases in confirmed cases, deaths, screening radius, and quarantine rate are associated with negative rewards for epidemic aggravating or causing economic burdens. Hence, the reward function is presented with a polynomial form:

7 $$PR\left({S}_{t}\right)= {E}_{t}-{a\times C}_{t}-{b\times D}_{t}-S-QR.$$

${S}_{t}$ is the probability of state at time step $t$. ${E}_{t}$ is the average economy. ${C}_{t}$ and ${D}_{t}$ are the daily confirmed cases and deaths, respectively. $S$ and $QR$ represent the average screening and quarantine rate. Parameters $a$ and $b$ are factitiously defined hyper-parameters and obtained from simulations.

During training, the average reward rises rapidly during the first 250 episodes and plateaus after 500 episodes, indicating the model is stable and the Agent does learn from interacting with the Environment to improve the policy. The detailed procedures of the Agent training are provided in Additional file 5.

### Simulating wave-specific environments

**Fig. 2:**
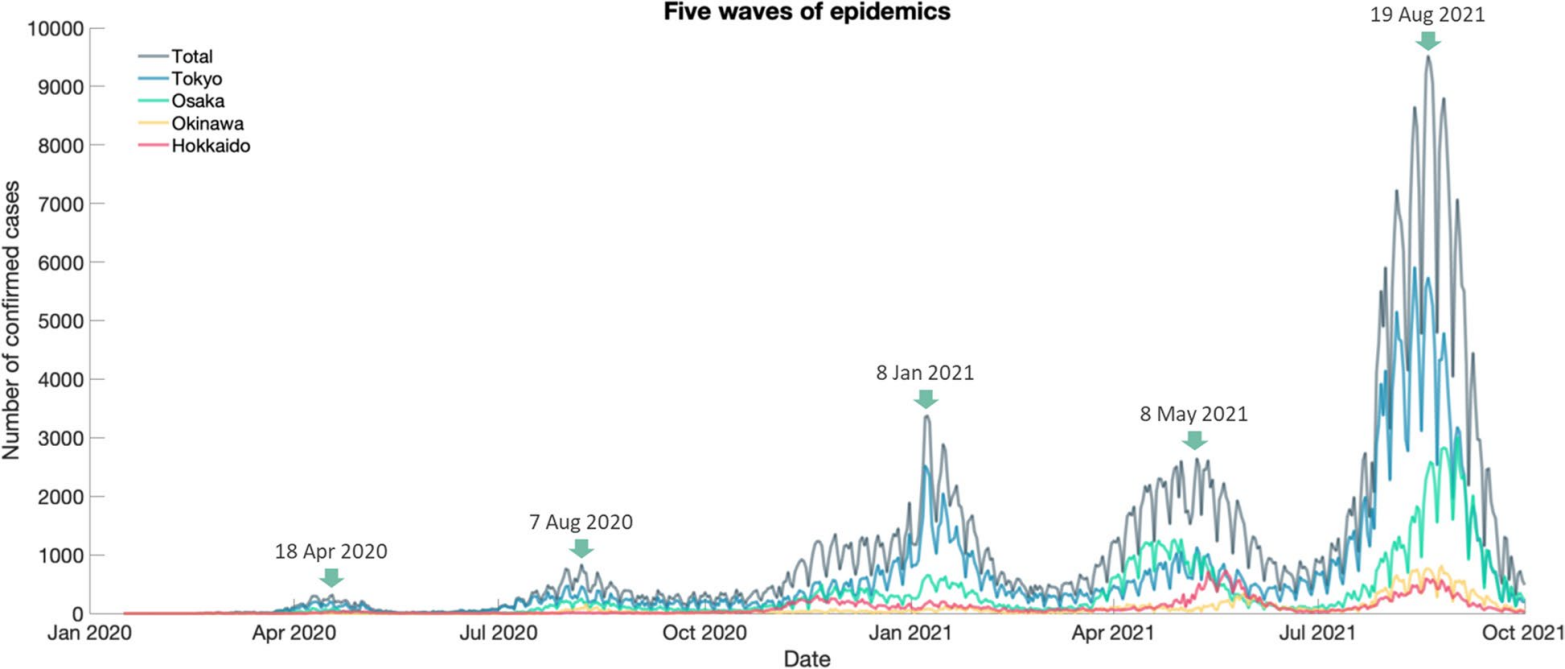
Five waves of epidemics. The green arrows indicate the dates of overall peaks

The trained Agent must be introduced into an Environment reflecting the real world to examine its performance. Five epidemic peaks are observed during the study period (Fig. 2). To simulate a trend like this, a compartmental model must be replenished with susceptible individuals; however, it would introduce model complexity and uncertainty. Alternatively, we treat each wave as a separate Environment. These 5 Environments have the same configuration as the one used for training except for daily limits of exposed cases as SEIQR model constraints. When the number of exposed cases reaches the limit, the transmission radius is reduced. To check whether the simulated Environments can represent the 5 waves, we set target numbers for the 4 prefectures using their confirmed case percentages at overall peaks (Table 2). The total number of infectious cases for the 5 overall peaks is arbitrarily set to be 300. We have a total of 2,500 individuals in the 5 Environments, but the population of the 4 prefectures is over 29 million. Hence, our intention is not to echo the actual incidences, which is not feasible under computational limitations, but to make the peak infectious cases proportionally match the peak confirmed cases across prefectures and epidemic waves. As shown in Table 2, the mean numbers of infectious cases from 10 simulations (see Additional file 6) are very close to the targets (the 5th and 4th rows of each peak), verifying that the simulated Environments coincide with the 5 waves.

**Table 2:** Calculation of wave-specific environment parameters

Peak	Parameters	Tokyo	Osaka	Okinawa	Hokkaido	Subtotal
**1st 4/18/2020**	Confirmed case	**186 (a)**	88	9	38	**321 (d1)**
	Percentage to total	**1.13% (b)**	0.53%	0.05%	0.23%	1.95%
	Limits of exposed case	2	1	1	1	5
	Target infectious case	**3 (c)**	2	0	1	6
	Mean simulated infectious case (SD)	3 (± 2)	1 (± 1)	1 (± 1)	1 (± 1)	6 (± 2)
	Infectious case with Agent’s involvement	0	2	1	1	4
**2nd 8/7/2020**	Confirmed case	461	255	100	14	**830 (d2)**
	Percentage to total	2.80%	1.55%	0.61%	0.08%	5.04%
	Limits of exposed case	4	3	3	0	10
	Target infectious case	8	5	2	0	15
	Mean simulated infectious case (SD)	8 (± 2)	5 (± 2)	1 (± 1)	1 (± 1)	15 (± 2)
	Infectious case with Agent’s involvement	0	4	2	0	6
**3rd 1/8/2021**	Confirmed case	2459	654	82	181	**3376 (d3)**
	Percentage to total	14.92%	3.97%	0.50%	1.10%	20.48%
	Limits of exposed case	28	7	1	1	37
	Target infectious case	45	12	1	3	61
	Mean simulated infectious case (SD)	48 (± 3)	7 (± 4)	0 (+ 1)	3 (± 2)	58 (± 4)
	Infectious case with Agent’s involvement	12	6	1	1	20
**4th 5/8/2021**	Confirmed case	1121	1020	93	403	**2637 (d4**)
	Percentage to total	6.80%	6.19%	0.56%	2.44%	16.00%
	Limits of exposed cases	8	15	1	3	27
	Target infectious cases	20	19	2	7	45
	Mean simulated infectious cases (SD)	24 (± 6)	15 (± 5)	2 (± 2)	7 (± 5)	48 (± 5)
	Infectious case with Agent’s involvement	5	3	0	10	18
**5th 8/19/2021**	Confirmed case	5534	2443	768	575	**9320 (d5)**
	Percentage to total	33.57%	14.82%	4.66%	3.49%	56.54%
	Limits of exposed case	59	33	11	10	113
	Target infectious case	101	44	14	10	165
	Mean simulated infectious case (SD)	98 (± 7)	46 (± 5)	14 (± 4)	8 (± 4)	166 (± 3)
	Infectious case with Agent’s involvement	19	10	5	1	35
**Total**						**16,484 (e)**

Total number of confirmed cases of 5 peaks (e) = (d1) + (d2) + (d3) + (d4) + (d5) = 16,484; Tokyo’s percentage to total at 1st peak (b) = (a) / (e) = 186 / 16,484 = 1.13%; Tokyo’s target infectious cases at 1st peak (c) = (b) * 300 = 1.13% * 300 ≈ 3. The means and SDs of simulated infectious cases are obtained from 10 simulations for each wave

## Results

**Fig. 3:**
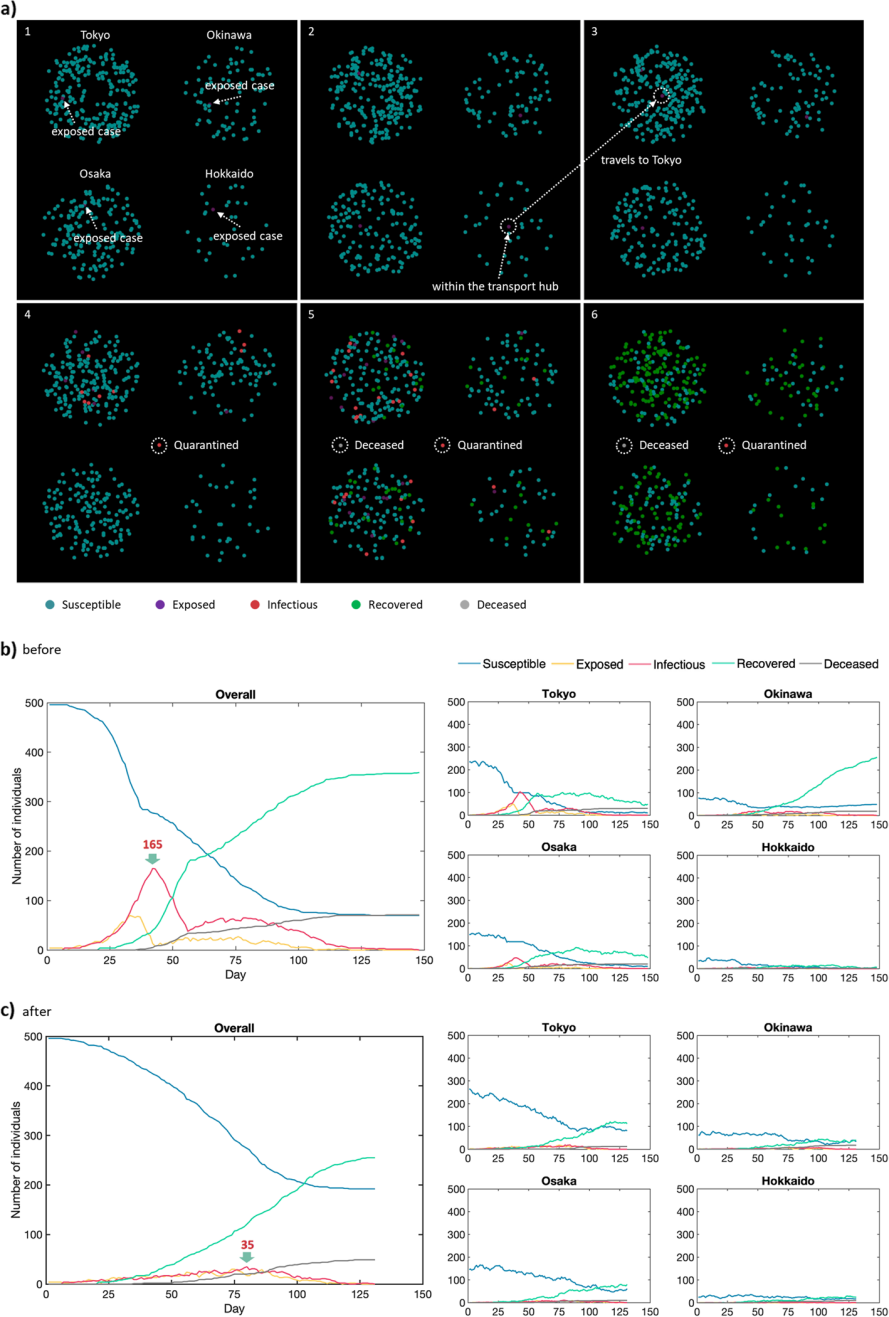
Simulation of the 5th wave of epidemic with and without the agent's involvement. **a** Simulation of the 5th epidemic wave using the Environment. **b** and **c** Overall and regional curves for the 5th wave before and after Agent’s engagement, respectively

Figure 3a elaborates on the SEIQR-RL simulation of the 5th wave. Figure 3a1 shows the initial state with one exposed case in each region. In Fig. 3a2, the exposed case of Hokkaido appears at the transport hub and then travels to Tokyo (Fig. 3a3). Quarantined and deceased cases will be displayed in the designated areas outside the 4 regions, and Fig. 3a4 and 3a5 exhibit the first quarantined and deceased cases surrounded by dashed circles, respectively. Figure 3a6 is the final state. An additional movie file shows the simulation (see Additional file 7). To compare the change before and after the Agent’s engagement, Fig. 3b displays the simulated overall and regional curves with no screening/quarantine or restriction on movement (i.e. screening radius = 0 pixels and moving distance = 5 pixels). However, with the trained Agent’s engagement as shown in Fig. 3c, the epidemic is shortened from 148 to 131 days, and the overall daily infectious maximum is dramatically reduced from 165 to 35. Deaths are also restricted from 71 to 49. Similar results are observed from the other 4 Environments (see Additional file 8), and the reduced peak numbers are provided in Table 2 at the 6th row of each peak. Simulated data with and without the Agent’s engagement for the 5 waves are provided in Additional files 9, 10, 11, 12, 13, 14, 15, 16, 17, 18.

**Fig. 4:**
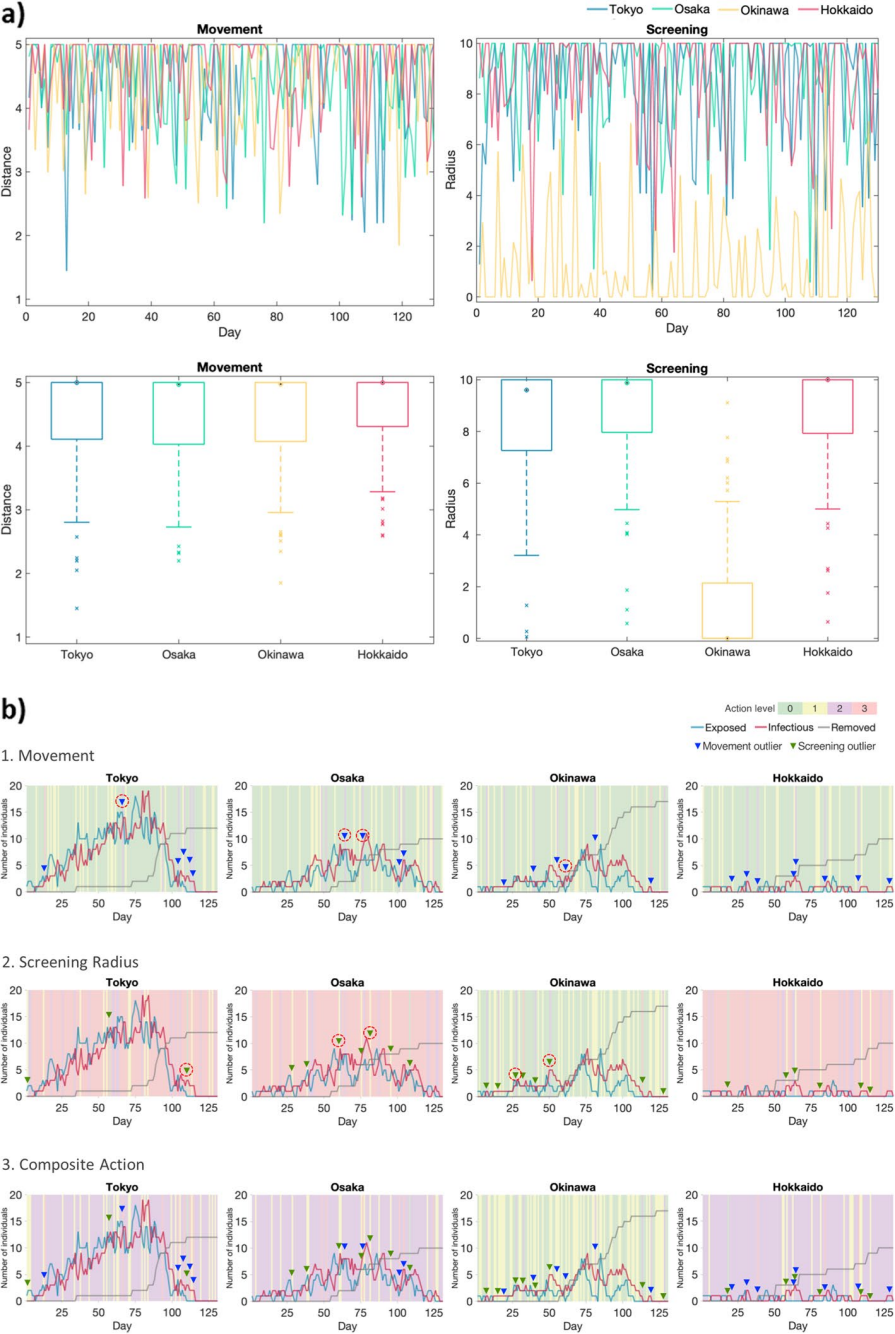
Action trends and action timing analyses of the 5th wave of the epidemic. **a** Action trends and boxplots of the simulated 5th epidemic. **b** Action timing analyses

Figure 4a shows the action trends and statistics of the 5th wave. For actions on movement, the medians are equal or close to 5, indicating minimal restrictions on movement for about half of the time. However, outliers are noticed. For actions on screening, on the other hand, the medians almost reach 10, suggesting the Agent frequently uses broad screening to control disease transmission. Yet the Agent acts differently on Okinawa; its median screening radius approaches 0. Similar results were observed from the other 4 waves (see Additional file 19).

To investigate action timings, we divide the action scores into 4 ordinal levels, with higher levels indicating more rigorous actions. Levels 0 to 3 represent moving distances of 4–5, 3–4, 2–3, and 1–2 pixels, or screening radiuses of 0–1 pixels, 1–5 pixels, 5–7 pixels, and 7–10 pixels. We also create composite action levels by adding up the action levels – level 0 for additions equal to 0, level 1 for 1–3, level 2 for 3–5, and level 3 for 5–6. Figure 4b depicts the time series analyses for the 5th wave, and action levels 0–3 are displayed with green, yellow, purple, and pink backgrounds, respectively. As indicated in Fig. 4a, actions are generally easy at levels 0 and 1 (green and yellow) on movements (Fig. 4b1) but more stringent at level 3 (pink) on screening (Fig. 4b2), except for Okinawa, where both actions are easy. As for composite actions shown in Fig. 4b3, Okinawa is mostly at levels 0 and 1; actions of levels 2 and 3 are rare. However, level 2 composite actions are seen most frequently for the other three prefectures.

Action outliers are marked with blue and green dots for movement and screening radius, respectively. Figure 4b1 demonstrates that the restriction on movement is elevated when the number of exposed or infectious cases remains high (e.g., Tokyo’s day 67 and Osaka’s day 65), or the number of exposed or infectious cases increases rapidly (e.g., Okinawa’s days 62 and Osaka’s day 77). On the contrary, Fig. 4b2 shows the screening is eased when the number of exposed cases drops to a regional low (e.g., Tokyo’s day 111 and Osaka’s day 61) or the number of infectious cases drops rapidly (e.g., Osaka’s days 82). For Okinawa, strengthened screenings occur when the number of exposed or infectious cases remains high (e.g., days 28 and 51).

## Discussion

We successfully expand the typically isolated and closed SEIR model to a semi-connected SEIQR system that accommodates subregions and integrates disease transmission, cross-regional traveling, and policies. To our knowledge, this is the first study attaining dynamic human flows between compartmental models through the mechanism of transport hubs. Spatial metapopulation models also have subpopulations pertinent to geographic or administrative regions, with each subpopulation having an SEIR model. However, metapopulation models use mobility matrices constructed from airline and commuter data to define the force of infection that determines the rate at which a susceptible individual within a specific region becomes exposed. Therefore, in these types of models, inter-regional movements cannot be observed because individuals do not really move between regions [9].

We use real data to establish wave-specific Environments with similar trends to the 4 prefectures. Therefore, factors that may affect disease transmission patterns like vaccination, multi-strains, and voluntarily preventive behaviors are reflected in the RL Environments to a certain extent. The fact that the trained Agent uses the optimal policy learned to generate similar results across the 5 Environments implies that the model is robust to the change of dynamic resulting from vaccination.

Variability in population crowdedness and human movement affects the transmission of an infectious disease [41]. PWD takes the distribution of populations in subareas into account, reflecting the density experienced by the average person in that region [42]. Thus, it is a good surrogate for crowdedness to allocate populations and human flows in synthetic Environments.

Screening and quarantine effectively prevent COVID-19 transmission [43] but consume resources and create economic burdens. Even though negative rewards are assigned in the proposed reward function, the Agent still relies heavily on screening to control spreading, confirming its effectiveness. This result is backed by Taiwan’s initial success in the pandemic contributed to the combination of testing, contact tracing, and quarantine [44, 45]. A key challenge of modeling is that interventions and human behaviors are often entangled with each other. Our RL Environments replicate real data and hence can be used to test the added effects of a specific intervention in addition to the background situation. In contrast, the Agent restricts movements only when transmission speeds up or remains high to maintain economic activities.

**Fig. 5:**
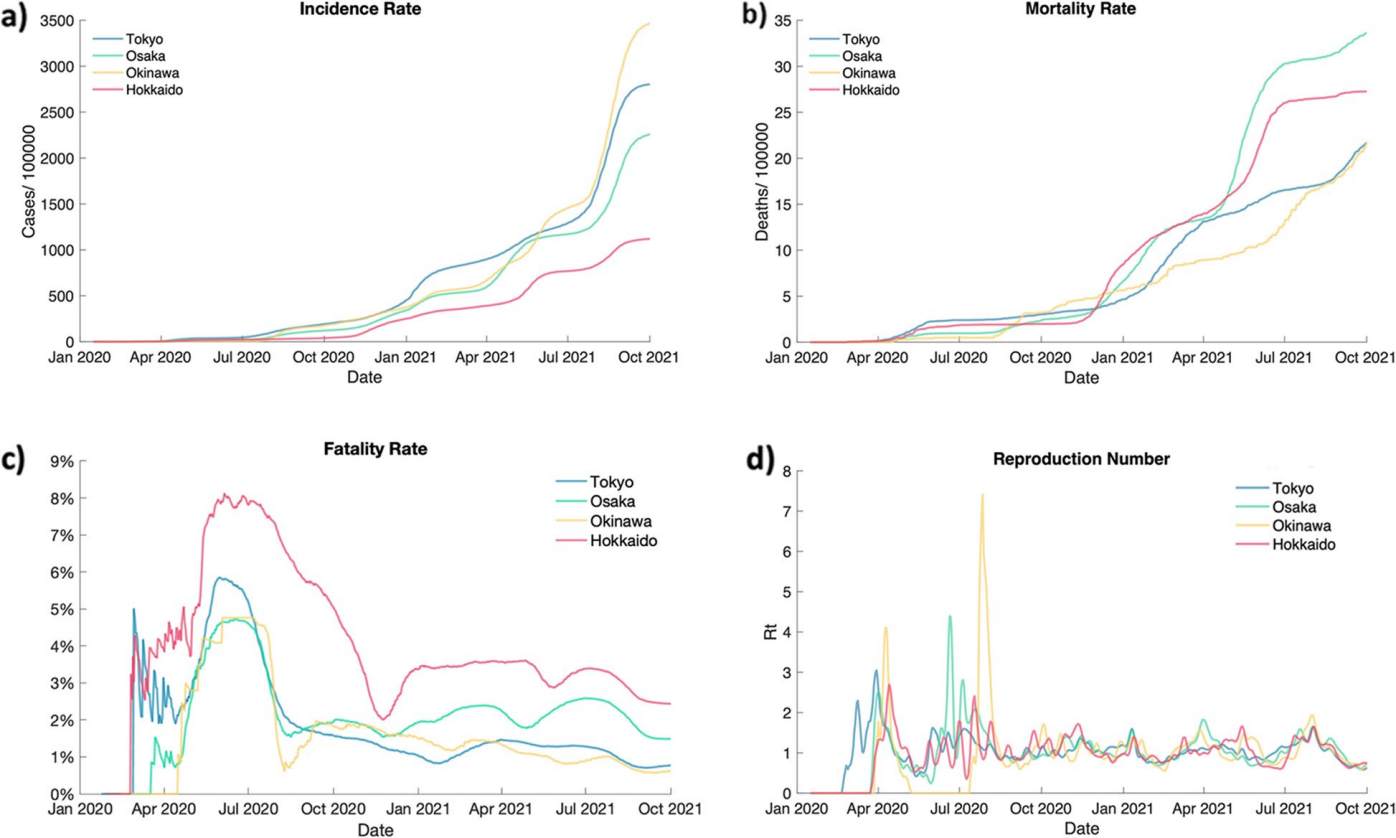
Incidence, mortality & fatality rates, and reproduction number. **a** Incidence rate, (**b**) mortality rate, (**c**) fatality rate, and (**d**) reproduction number (five-day moving average) of the study period. The rates are calculated assuming each person is only infected once

Deviated action patterns are observed in Okinawa. Over the study period, Okinawa has a high incidence rate and reproduction number (Fig. 5a and d), but low mortality and fatality rates (Fig. 5b and c). Since our Environments reflect the natural COVID-19 spreading scenes, we infer that the Agent treats Okinawa as a low-risk region partly because of these features and is supported by minimal movement restriction.

The results of our experiments should be interpreted given the following limitations. Firstly, only 500 individuals are allowed in each RL Environment due to computational constraints; subsequently, an excessive fatality rate of 20% is set for the SEIQR models to generate deaths. The results would be more representative and generalizable if the population could be increased by improving computational resources. Moreover, vaccination dynamics are not incorporated in our model; therefore, the results of the 4th and the 5th waves should be interpreted with caution as the Agent might take different strategies if vaccination effects were taken into account. Furthermore, actions are updated every 24 h. It is not feasible for authorities to issue new policies this frequently, but our algorithm still provides valuable information for reference in making policies. Lastly, our algorithm does not include international traveling, which could be ignored because border control was implemented in Japan during the experimental period. However, applying the model to other periods when international entries are allowed may affect the model's validity. Despite these limitations, our RL algorithm offers a nice container to observe the balance between disease control and economic activities and may assist in policy making.

## Conclusions

Our semi-connected SEIQR models establish an interactive environment that offers a nice container to observe the balance between disease control and economic activities. It also exhibits the potential of RL algorithms in supporting policy-making.

### Supplementary Information

**Supplementary Material 1.**

**Supplementary Material 2.**

**Supplementary Material 3.**

**Supplementary Material 4.**

**Supplementary Material 5**

**Supplementary Material 6.**

**Supplementary Material 7.**

**Supplementary Material 8.**

**Supplementary Material 9.**

**Supplementary Material 10.**

**Supplementary Material 11.**

**Supplementary Material 12.**

**Supplementary Material 13.**

**Supplementary Material 14.**

**Supplementary Material 15.**

**Supplementary Material 16.**

**Supplementary Material 17.**

**Supplementary Material 18.**

**Supplementary Material 19.**

## Data Availability

The datasets supporting the conclusions of this article are included within the article and its additional files.

## Abbreviations

A3C: Asynchronous Advantage Actor-Critic
COVID-19: Coronavirus Disease of 2019
GAE: Generalized Advantage Estimation
LSTM: Long Short-Term Memory
ML: Machine Learning
PD: Population Density
PPO: Proximal Policy Optimization
PWD: Population-Weighted Density
RL: Reinforcement Learning
R_t_: Reproduction Rate at Time t
SEIR: Susceptible-Exposed-Infected-Removed
SEIQR: Susceptible-Exposed-Infected-Quarantined-Removed
SIR: Susceptible-Infected-Removed
