# Supplementary figures and images for: A dynamic approach to support outbreak management using reinforcement learning and semi-connected SEIQR models
Source: BMC Public Health. 2024 Mar 11;24:751. doi: 10.1186/s12889-024-18251-0 (PMC10926678; doi:10.1186/s12889-024-18251-0)

## Slide 1
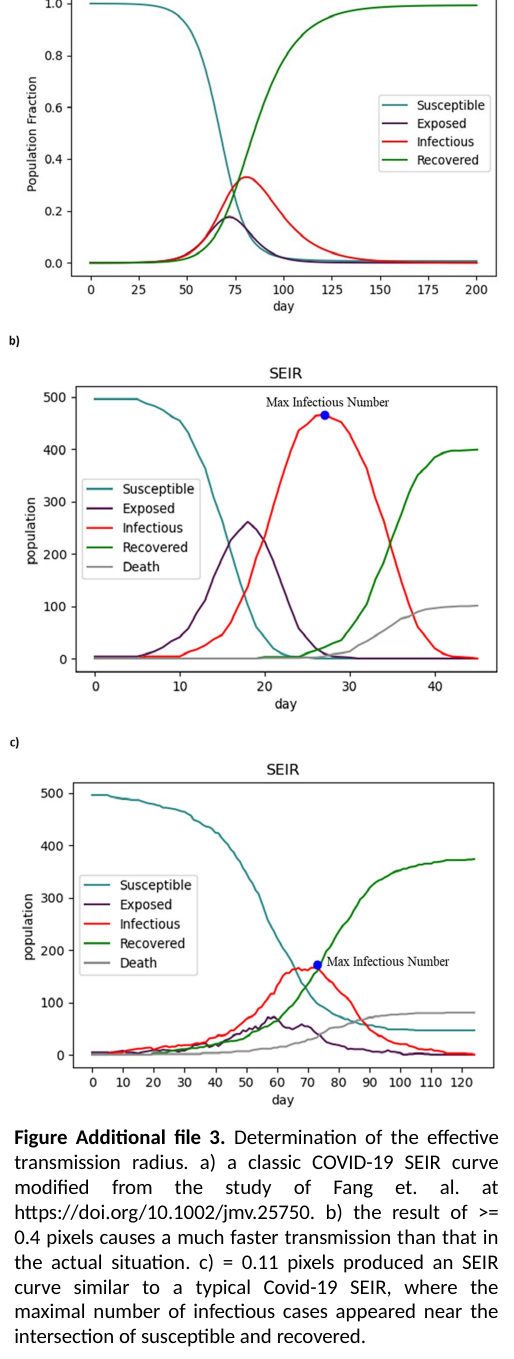

Supplement: Supplementary file 3 — Supplementary Material 3. [file 12889_2024_18251_MOESM3_ESM.pptx]

## Slide 1
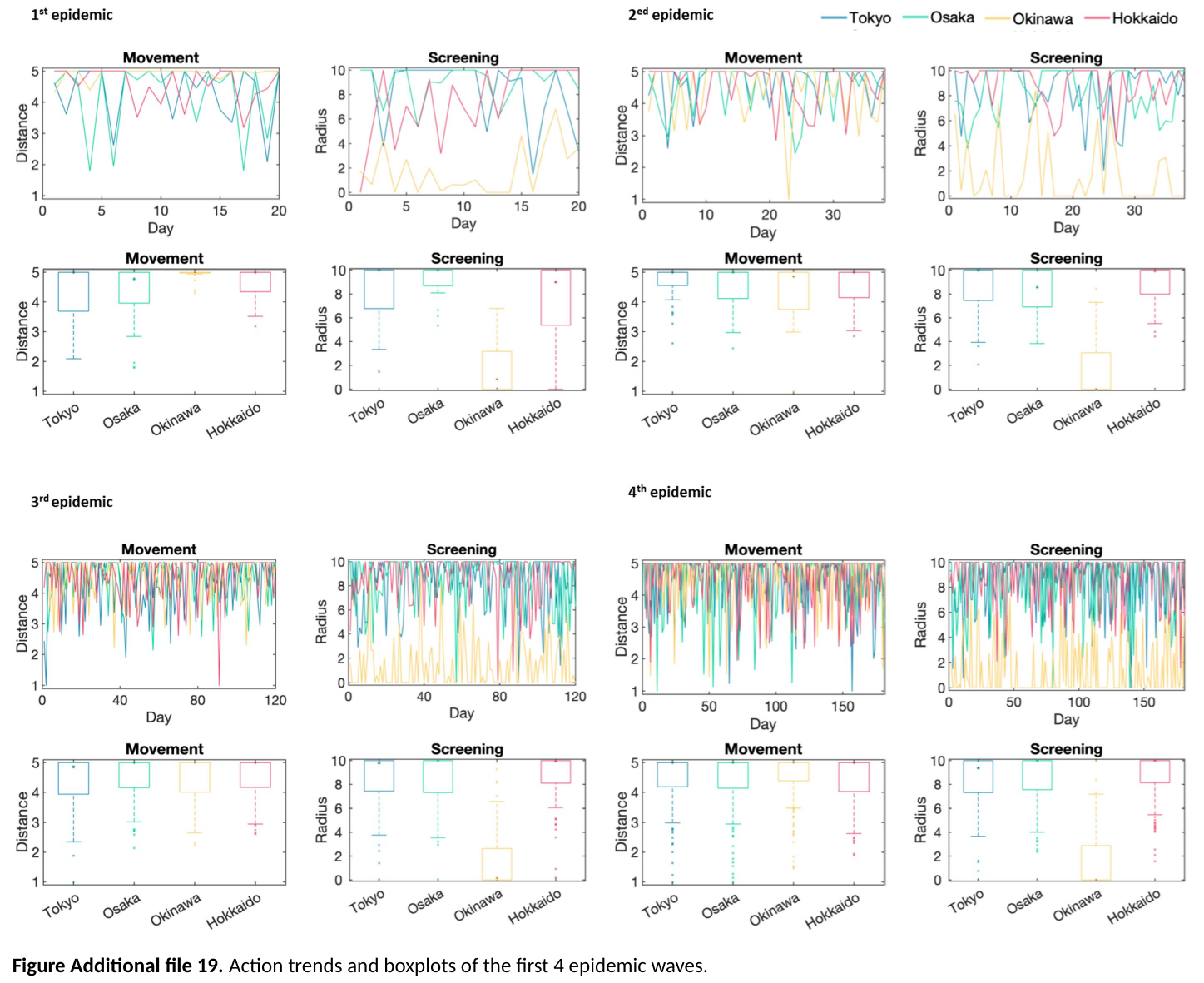

Figure Additional file 19. Action trends and boxplots of the first 4 epidemic waves.

Supplement: Supplementary file 19 — Supplementary Material 19. [file 12889_2024_18251_MOESM19_ESM.pptx]
