# Supplementary material for: A dynamic approach to support outbreak management using reinforcement learning and semi-connected SEIQR models
Source: BMC Public Health. 2024 Mar 11;24:751. doi: 10.1186/s12889-024-18251-0 (PMC10926678; doi:10.1186/s12889-024-18251-0)
Supplement: Supplementary file 4 — Supplementary Material 4. [file 12889_2024_18251_MOESM4_ESM.pptx]

## Slide 1
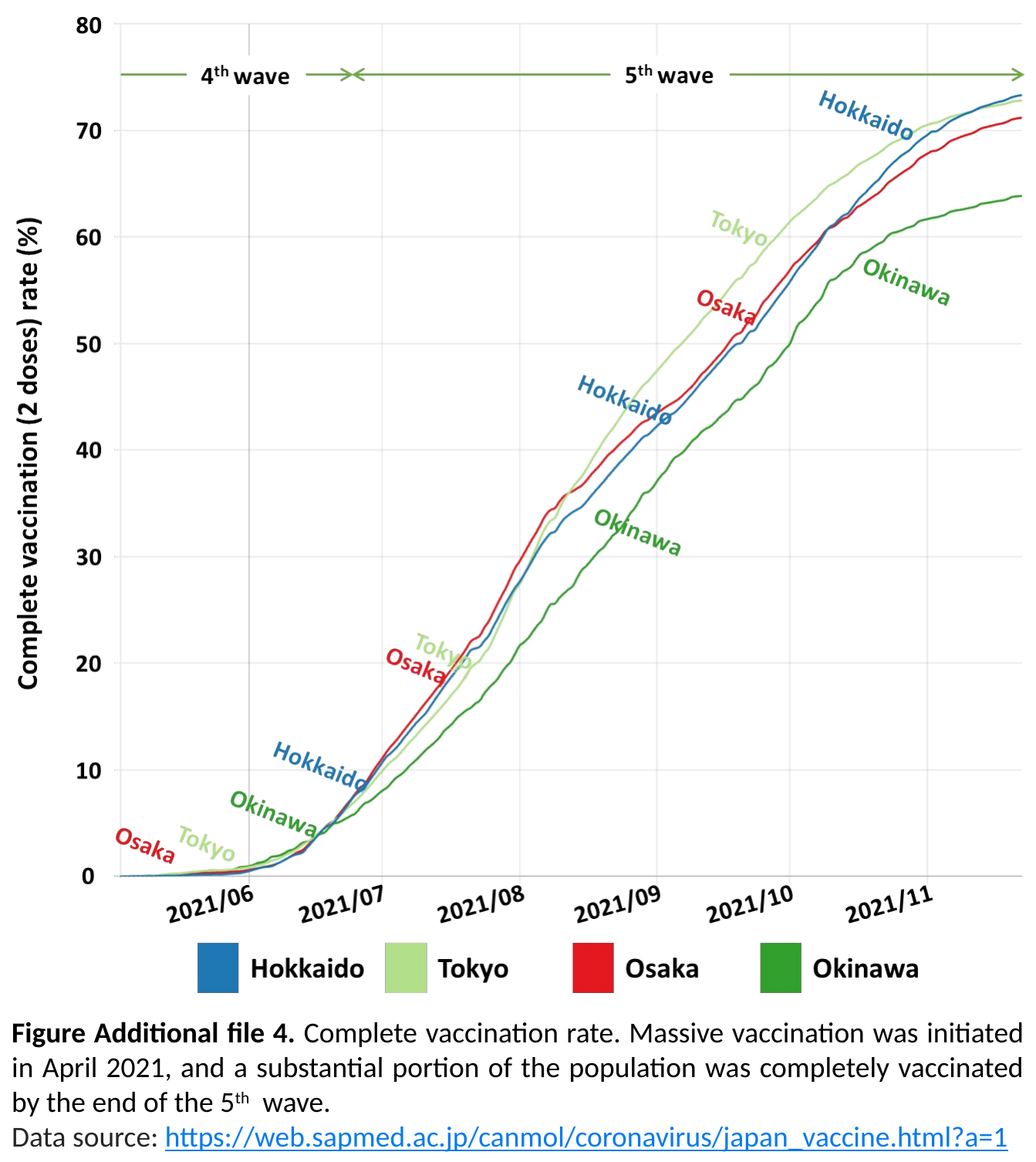

Figure Additional file 4. Complete vaccination rate. Massive vaccination was initiated in April 2021, and a substantial portion of the population was completely vaccinated by the end of the 5th wave.
Data source: https://web.sapmed.ac.jp/canmol/coronavirus/japan_vaccine.html?a=1
