# Supplementary material for: A dynamic approach to support outbreak management using reinforcement learning and semi-connected SEIQR models
Source: BMC Public Health. 2024 Mar 11;24:751. doi: 10.1186/s12889-024-18251-0 (PMC10926678; doi:10.1186/s12889-024-18251-0)
Supplement: Supplementary file 5 — Supplementary Material 5 [file 12889_2024_18251_MOESM5_ESM.docx]

1. **Agent design and training**

As mentioned in the manuscript, the agent granted two types of actions: movement and screening radius. These actions were in a continuous value domain with movements ranging from 1 to 5 pixels and screening radiuses ranging from 0 to 10 pixels and were executed at the prefectural level in the SEIQR environments. For instance, if the agent granted Tokyo a movement of 5 pixels and a screening radius of 10 pixels, these actions applied to all individuals in Tokyo. Different actions could apply to different regions. In all SEIQR environments, each screening area overlapped with the central station (the transport hub) and shares the exact center. The screening radiuses equal to 0, 5, and 10 pixels simulated the circumstances with no screening, in the station, and in and around the station, respectively.

Fig. 1 depicts the deep neural network structure employed for the environment training. The first part comprises three bidirectional long short-term memory (LSTM, please also refer to Fig. 2) layers, and each layer has 128, 64, and 64 nodes, respectively. The second part has three fully connected layers with 128, 64, and 32 nodes. Then, the rest has an output layer, with 4 nodes for movement and 4 for screening radius. In the LSTM block, the symbols $f$, $i$, $g$, and $o$ represent the forget gate, input gate, modulation gate, and output gate, respectively. Then, we used the sigmoid, tanh, and ReLU functions for the signal activation. The vector parameters $\boldsymbol{v}$, $\boldsymbol{w}$, and $\boldsymbol{u}$ are the weighting of cell state $c_{t-1}$, hidden state $h_{t-1}$, and the observed value $x_{t}$, respectively. Symbol $\hat{y}_{t}$ represents the predictive value and will be the input of the fully connected layer. The operators $⨀$ and $\text{⨁}$ are Hadamard product and elementwise addition, respectively. The operational procedures are defined as:

| $\left[ \begin{aligned} f \\ i \\ g \\ o \end{aligned} \right]=\left[ \begin{aligned} \begin{aligned} \text{sig}\left( \boldsymbol{w}_{f}h_{t-1}\text{⨁}\boldsymbol{u}_{f}x_{t} \right) \\ \text{sig}\left( \boldsymbol{w}_{i}h_{t-1}\text{⨁}\boldsymbol{u}_{i}x_{t} \right) \end{aligned} \\ \text{tanh}\left( \boldsymbol{w}_{g}h_{t-1}\text{⨁}\boldsymbol{u}_{g}x_{t} \right) \\ \text{sig}\left( \boldsymbol{w}_{o}h_{t-1}\text{⨁}\boldsymbol{u}_{o}x_{t} \right) \end{aligned} \right],$  $c_{t}=f⨀c_{t-1}+i⨀g,$  and  $h_{t}=o ⨀\text{ tanh}\left( c_{t} \right).$ | (1) |
| --- | --- |

The bidirectional LSTM was utilized to observe the time serial data, and it was anticipated that the agent could learn from the consecutive data and use them as input in its training procedure to generate proper actions.

Methods of asynchronous advantage actor-critic (A3C) [1], proximal policy optimization (PPO) [2], and generalized advantage estimation (GAE) [3] were used in training the agent. The actor-critic methods are popular RL algorithms integrating policy-based (training an actor) and value-based (training a critic) approaches. The actor learns to conduct actions while the critic assesses value functions to assist the actor with learning. In our A3C framework shown in Fig. 3, 18 auxiliary agents parallelly interact with their corresponding environments and pass parameters to the primary agent to generate global parameters. The actor and critic use the same network structure and inputs (states), but their outputs differ. The actor outputs the mean and standard deviation of normal distributions for action selections. The critic, on the other hand, evaluates the actor's performance. Based on parallel computing, A3C increases training data diversity and achieves faster updates through batch agent learning to reach a common goal.

We then employed the PPO technique as our optimization algorithm for policy gradients. The clipped objective function of PPO is

| $L\left( \theta\right)\mathbb{=E}\left[ \min\left( r_{t}\left( \theta\right)A^{\pi_{\theta}}, \mathrm{clip}\left( r_{t}\left( \theta\right), 1-\epsilon,1+\epsilon\right)A^{\pi_{\theta}} \right) \right],$ | (2) |
| --- | --- |

where $\theta$ is the policy parameter, $\epsilon$ is a hyper-parameter, and $A^{\pi_{\theta}}$ is the estimated advantage. Symbols $\mathbb{E}$ is an expectation operator. Function $r_{t}\left( \theta\right)$ is the ratio of the probabilities between the new policy $\pi_{\theta}\left( \tau\right)$ and previous policy $\pi_{\theta_{pre}}\left( \tau\right)$ with the corresponding action $\tau$:

| $r_{t}\left( \theta\right)=\frac{\pi_{\theta}\left( \tau\right)}{\pi_{\theta_{pre}}\left( \tau\right)}.$ | (3) |
| --- | --- |

The PPO would limit the $r_{t}\left( \theta\right)$ values within the range of $\left( 1-\epsilon,1+\epsilon\right)$ to ensure the updated policy is not too different from the previous policy. The consequence leads to less variance and more accuracy in agent training.

Furthermore, we utilized the GAE technique to reduce the variance in policy gradient estimation. Methods of temporal difference (TD) and Monte Carlo (MC) are conventional estimation methods frequently used in RL. The TD method could offer lower variance in training procedures but also introduce bias. On the contrary, the estimations of MC are relatively precise. But it would also cause higher variances in training. Thus, we employed the GAE technique to balance these two extremes to provide reliable and accurate estimations with less variance. As illustrated in Fig. 4, the average reward rose rapidly during the first 250 episodes and plateaus after 500 episodes, indicating the agent learned well from the RL environment and the model was stable. The hardware of the RL environment and agent training was CPU Intel Core i9-10980XE with 18 cores and 36 threads GPU GeForce RTX 3090 2ith 24GB. There were 18 workers for multi-threading on the A3C technique (see Fig. 3).

1. **Design of reward function**

An RL agent learns the optimal policy via a reward function. The agent would favor the actions that increase rewards. In our experiment, promoting economic activities was designed to generate positive rewards. On the contrary, the number of infectious cases and deaths was reduced with negative rewards. Screening radius and quarantine rate also caused negative rewards for increasing economic burdens. Thus, our reward function $PR\left( s_{t} \right)$ was designed as a combination of the above viewpoints and presented with a polynomial form (please also refer to Eq. (7) of the manuscript):

| $PR\left( s_{t} \right)= E_{t}-{a\times I}_{t}-{b\times D}_{t}-SR-QR,$ | (4) |
| --- | --- |

where $a=800$ and $b=5$ are hyper-parameters, and $s_{t}$ is the probability of state at time-step $t$. The other variables are

| $E_{t}=\frac{Daily total movement}{\left( Total Population \right)\times\max\left( movement \right)},$ | (5) |
| --- | --- |
| $I_{t}=\frac{DI}{Total Population},$ | (6) |
| $D_{t}=\frac{DD}{Total Population},$ | (7) |
| $SR=\frac{\sum RS in each prefectures}{\max\left( RS \right)},$  and | (8) |
| $QR=\frac{\max\left( \left[ DQ-\max\left( QA \right) \right], 0 \right)}{\max\left( QA \right)}.$ | (9) |

The symbols $DI$, $DD$, and $DQ$ represent the cases of daily infectious, daily death, and daily quarantine, respectively. Then the $RS$ and $QA$ mean the radius of screening and the human capacity of the quarantine area, respectively. To remove the influence of units, all variables were presented as percentages.

**
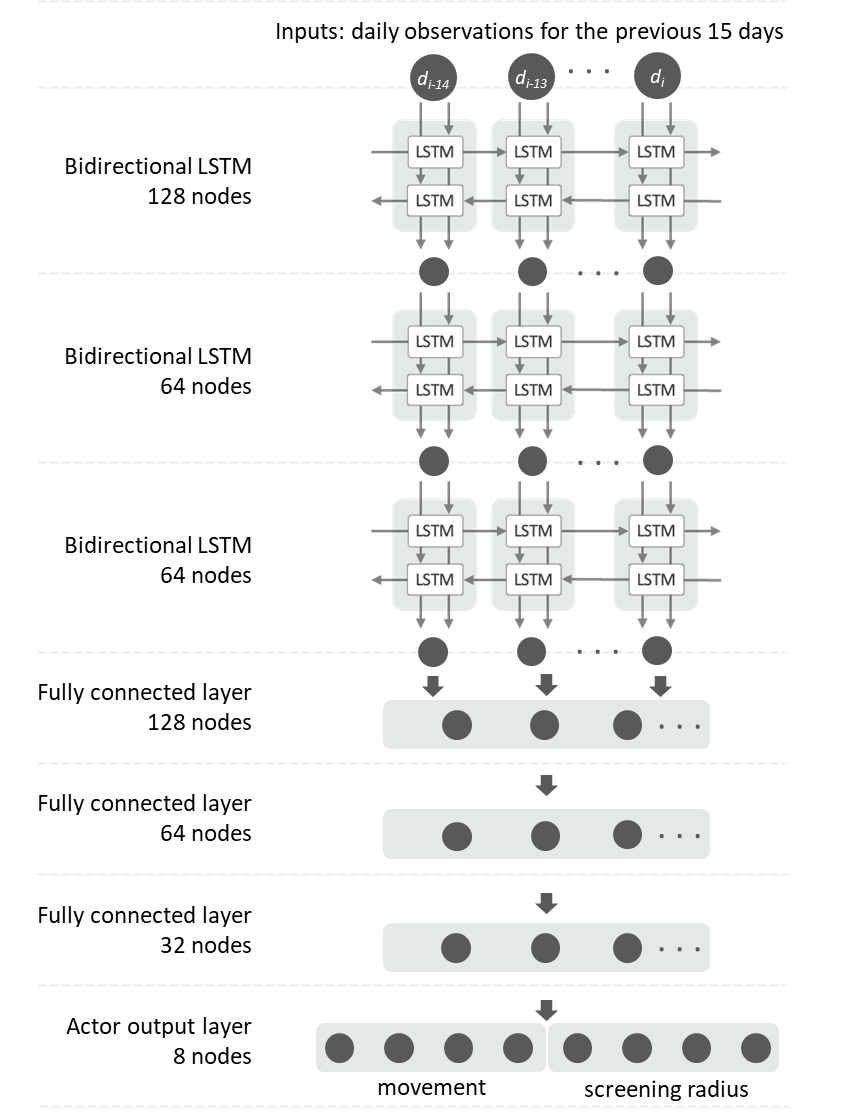
**

**Figure 1.** Employed network for RL environment training.


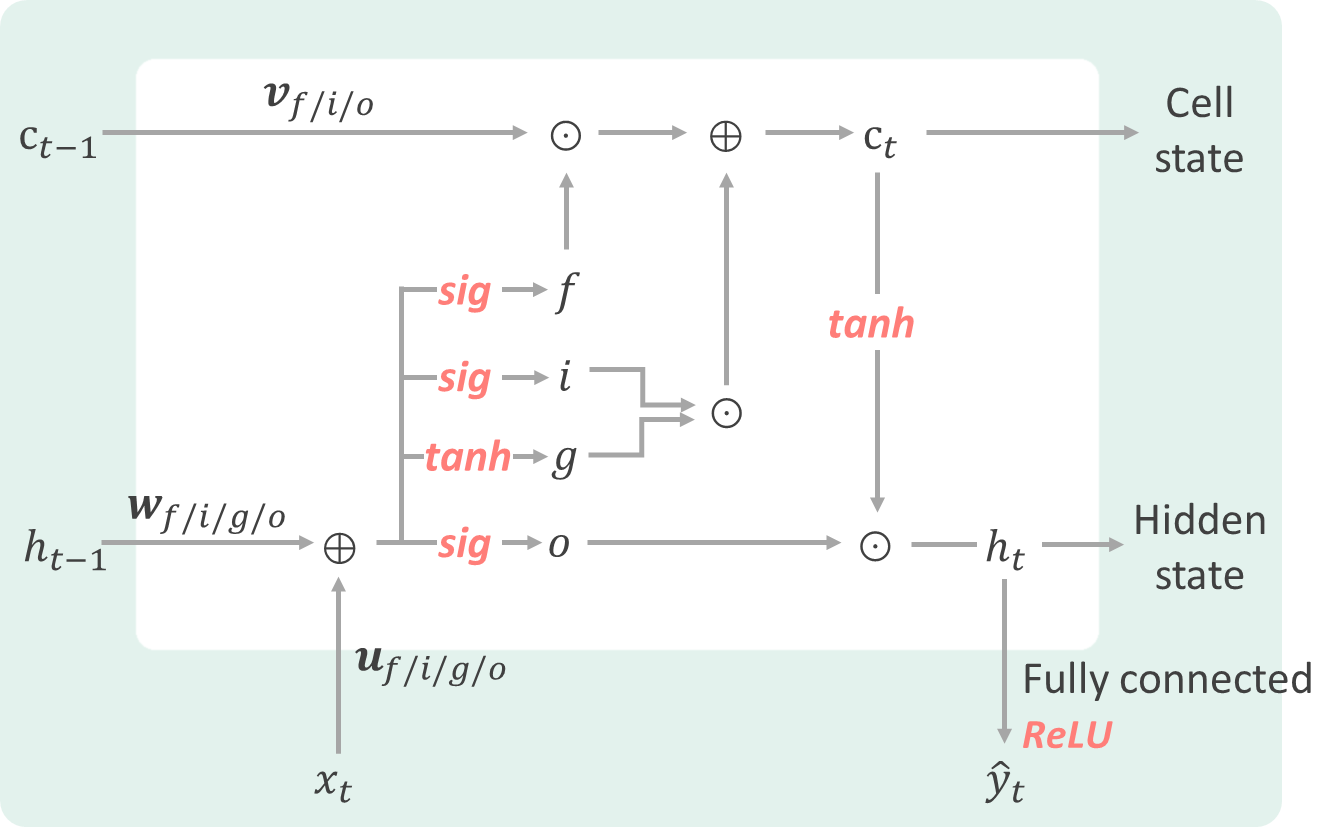


**Figure 2.** Scheme of the LSTM block.

**
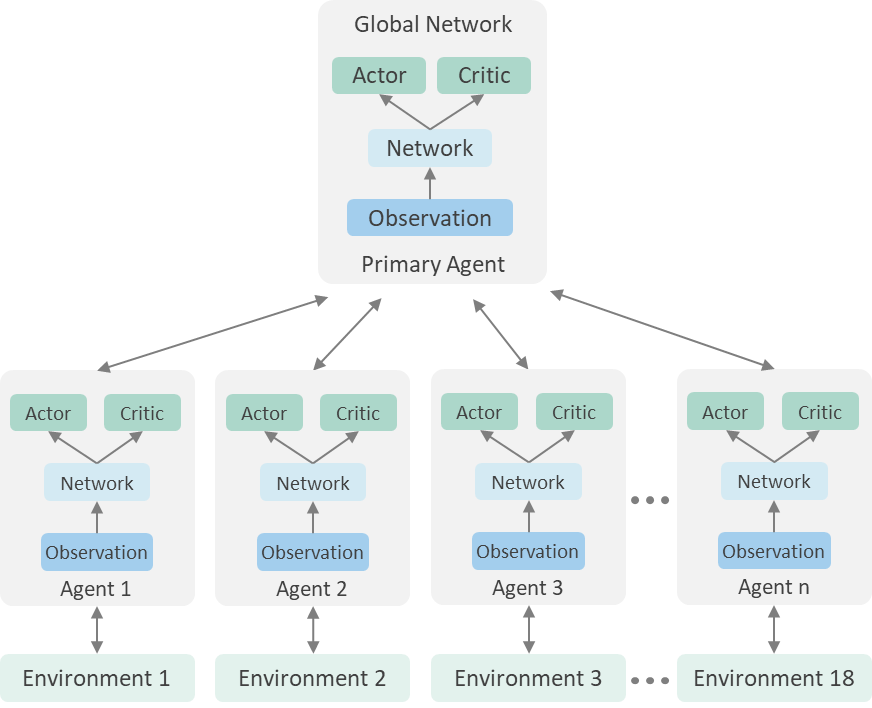
**

**Figure 3.** Structure of the A3C in the study.


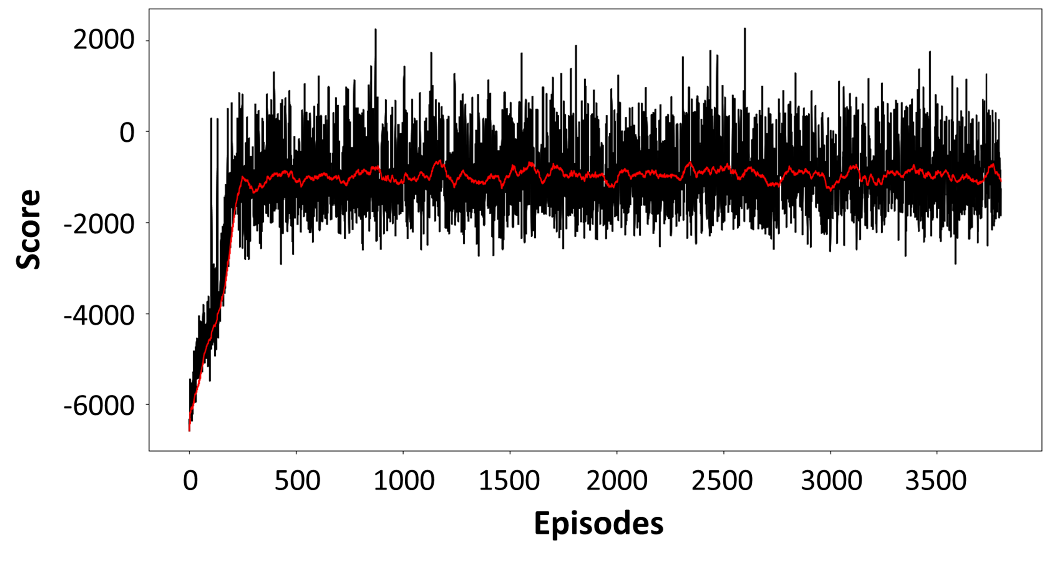


**Figure 4.** The trend of the average reward during training.

**Reference**

1. M. Babaeizadeh, I. Frosio, S. Tyree, J. Clemons, and J. Kautz, "Reinforcement learning through asynchronous advantage actor-critic on a gpu," arXiv preprint, 2016, doi: 10.48550/arXiv.1611.06256.
2. Y. Yu, X. Si, C. Hu, and J. Zhang, "A review of recurrent neural networks: LSTM cells and network architectures," Neural computation, vol. 31, no. 7, pp. 1235-1270, 2019.
3. M. J. Kochenderfer, T. A. Wheeler, and K. H. Wray, Algorithms for decision making. MIT Press, 2022.
