# Supplementary material for: A dynamic approach to support outbreak management using reinforcement learning and semi-connected SEIQR models
Source: BMC Public Health. 2024 Mar 11;24:751. doi: 10.1186/s12889-024-18251-0 (PMC10926678; doi:10.1186/s12889-024-18251-0)
Supplement: Supplementary file 7 — Supplementary Material 7. [file 12889_2024_18251_MOESM7_ESM.pptx]

## Slide 1
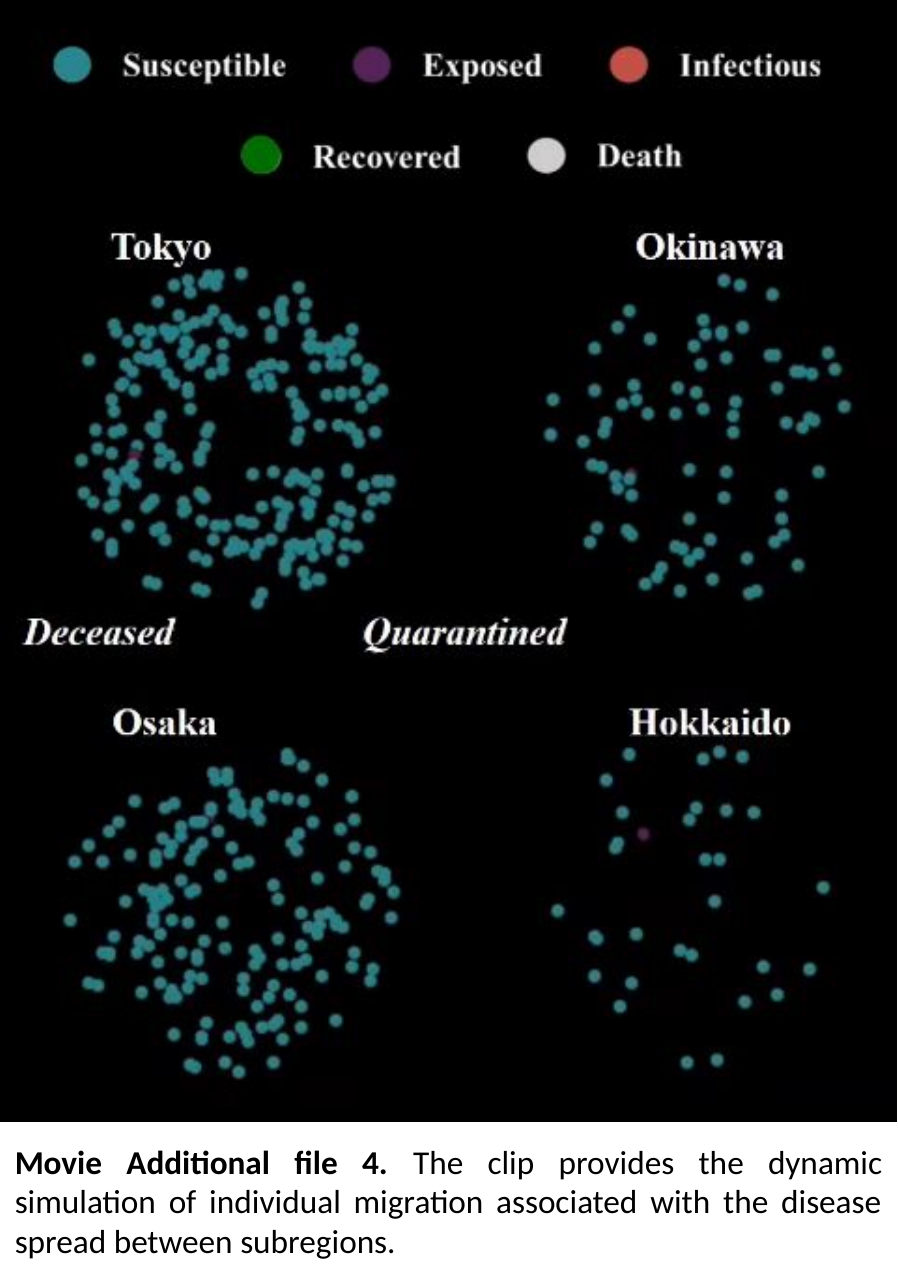

Movie Additional file 4. The clip provides the dynamic simulation of individual migration associated with the disease spread between subregions.
