# Supplementary material for: A dynamic approach to support outbreak management using reinforcement learning and semi-connected SEIQR models
Source: BMC Public Health. 2024 Mar 11;24:751. doi: 10.1186/s12889-024-18251-0 (PMC10926678; doi:10.1186/s12889-024-18251-0)
Supplement: Supplementary file 8 — Supplementary Material 8. [file 12889_2024_18251_MOESM8_ESM.pptx]

## Slide 1
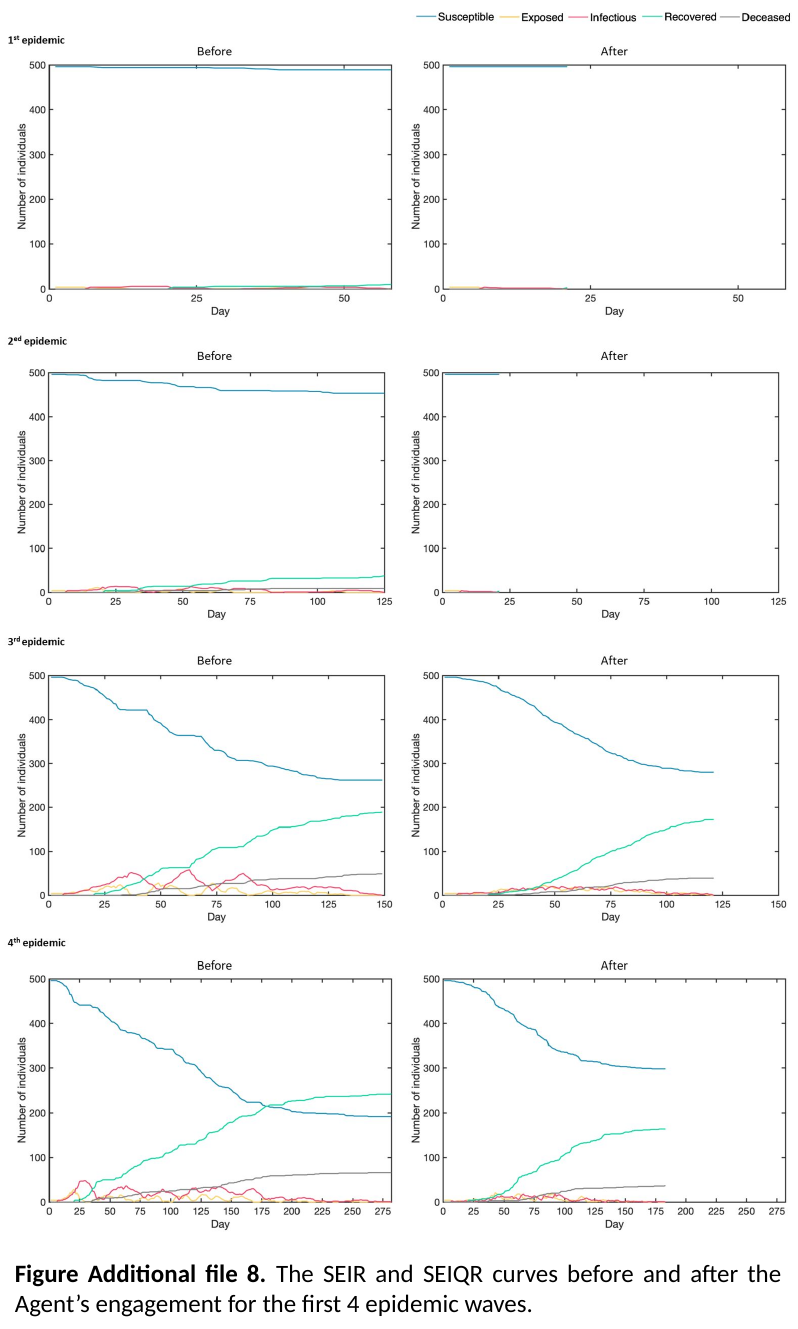

Figure Additional file 8. The SEIR and SEIQR curves before and after the Agent’s engagement for the first 4 epidemic waves.
